# *MYBPC1*-associated congenital myopathy with tremor: further delineation of the clinical and pathological phenotype in the first Italian case

**DOI:** 10.3389/fgene.2026.1809063

**Published:** 2026-05-14

**Authors:** Daniele Velardo, Claudia Alberti, Delia Gagliardi, Roberto Del Bo, Patrizia Ciscato, Laura Napoli, Simona Zanotti, Michela Ripolone, Michele Giovanni Croce, Giuseppe Cosentino, Gabriele Tumminello, Marco Locatelli, Giacomo Pietro Comi, Stefania Corti, Sabrina Ravaglia, Dario Ronchi

**Affiliations:** 1 Neuromuscular and Rare Disease Unit, Fondazione IRCCS Ca’ Granda Ospedale Maggiore Policlinico, Milan, Italy; 2 Department of Pathophysiology and Transplantation, Dino Ferrari Center, University of Milan, Milan, Italy; 3 Neurology Unit, IRCCS Fondazione Ca’ Granda Ospedale Maggiore Policlinico, Milan, Italy; 4 Department of Brain and Behavioural Sciences, University of Pavia, Pavia, Italy; 5 Translational Neurophysiology Research Unit, IRCCS Mondino Foundation, Pavia, Italy; 6 Department of Cardio-Thoracic-Vascular Diseases, IRCCS Fondazione Ca’ Granda Ospedale Maggiore Policlinico, Milan, Italy; 7 Unit of Neurosurgery, Fondazione IRCCS Ca’ Granda Ospedale Maggiore Policlinico of Milan, Milan, Italy; 8 Department of Medical-Surgical Physiopathology and Transplantation, University of Milan, Milan, Italy; 9 Neuromuscular Unit, IRCCS Mondino Foundation, Pavia, Italy

**Keywords:** case report, congenital myopathy, MYBPC1, neuromuscular disorder, tremor

## Abstract

The *MYBPC1* gene, mapping to chromosome 12q23.2, encodes the slow myosin binding protein-C (sMyBP-C), a sarcomeric accessory protein, expressed mainly in slow skeletal muscle fibers, that aids in the regulation of actomyosin cross-bridges and provides thick filament stability. Biallelic molecular defects in *MYBPC1* are associated with a lethal congenital form of myopathy (Lethal congenital contracture syndrome 4); meanwhile, heterozygous damaging variants lead to a form of distal arthrogryposis (distal arthrogryposis type 1B) and an early‐onset congenital myopathy with tremor (congenital myopathy-16, CMYO16). To date, only 20 cases have been documented, all presenting a mild axial–proximal myopathy consistently coupled with a distinctive tremor phenotype. We report the case of a young Italian woman presenting with mild axial and proximal weakness, associated with a high-frequency postural tremor affecting the limbs and tongue, with apparent exacerbation of symptoms after hormonal stimulation. Laboratory tests showed normal creatine kinase levels. Electromyography revealed diffuse mild myopathic changes. Muscle MRI was substantially normal. Polygraphic tremor analysis confirmed the presence of a postural tremor at a frequency of 10–11 Hz. Muscle biopsy showed selective type 1 fiber hypotrophy. Clinical exome sequencing revealed the heterozygous c.788T>G p.(Leu263Arg) variant in exon 11 in the *MYBPC1* gene. This variant has been previously reported in multiple independent subjects displaying skeletal muscle weakness and myogenic tremor. Our case helps further define the phenotypic spectrum of this disorder, providing additional clinical and pathologically relevant insides, including the possible influence of hormonal stimulation and a detailed characterization of muscle biopsy findings. Knowledge of this myopathic phenotype may allow identification of individuals with *MYBPC1* variants without arthrogryposis.

## Introduction

Congenital myopathies are characterized by marked phenotypic and genetic heterogeneity. Alterations in a single sarcomeric protein can result in a wide range of clinical presentations, from early-onset to adult-onset, causing either reduced muscle strength or stiffness.

Myosin-binding protein C (MyBP-C) constitutes a class of accessory sarcomeric proteins that play critical roles in modulating actomyosin cross-bridge interactions and in maintaining the structural stability of thick filaments within striated muscle fibers. The family comprises three isoforms—slow skeletal (sMyBP-C), fast skeletal (fMyBP-C), and cardiac (cMyBP-C)—encoded by *MYBPC1*, *MYBPC2*, and *MYBPC3*, respectively (MIM#160794, 160,793, and 600,958) (Ackermann and Kontrogianni-Konstantopoulos, 2010; McNamara and Sadayappan, 2018; Weber et al., 1993). Whereas cMyBP-C is specifically expressed in cardiac muscle, fMyBP-C and sMyBP-C are co-expressed in both fast- and slow-twitch skeletal muscles in varying proportions (Ackermann and Kontrogianni-Konstantopoulos, 2011).

sMyBP-C is a modular protein composed of seven immunoglobulin (Ig) domains and three fibronectin type III (Fn-III) domains, designated C1 through C10, interspersed with distinctive sequence segments. These include a proline/alanine-rich region and a MyBP-C–specific segment known as the M-motif, which is positioned adjacent to the C1 Ig domain (Ackermann and Kontrogianni-Konstantopoulos, 2013).

**FIGURE 1:**
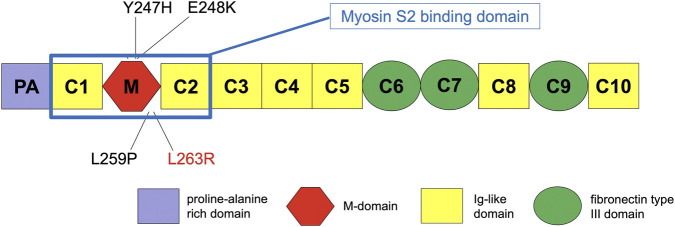
Pathogenic variants in MYBPC1. Schematic representation of the domain structure of slow skeletal MyBP-C (sMyBP-C) encoded by MYBPC1 and depiction of known pathogenic variants associated with congenital myopathy with tremor. MYBPC1 is unique among the MYBPC genes as it is heavily spliced, giving rise to at least 14 known isoforms in humans encoding proteins between 126 and 131.5 kDa.

The *MYBPC1* gene, located on chromosome 12q23.2, is distinctive within the MYBPC family in that it undergoes extensive alternative splicing, producing a subfamily of at least fourteen known human isoforms that encode proteins ranging from approximately 126–131.5 kDa (Figure 1). These isoforms are co-expressed in various combinations and relative abundances across both slow- and fast-twitch skeletal muscle fibers (Ackermann and Kontrogianni-Konstantopoulos, 2013). In addition, the phosphorylation profile of sMyBP-C in mouse and human fast-twitch skeletal muscles is differentially regulated by aging and disease, indicating a key role for phosphorylation in these processes. (Ackermann et al., 2015).

Biallelic molecular defects in *MYBPC1* are associated with a lethal congenital form of myopathy (Lethal congenital contracture syndrome 4, MIM 614915) (Markus et al., 2012) and Arthrogryposis Multiplex Congenital (Ekhilevitch et al., 2016). Conversely, heterozygous damaging variants, occurring *de novo* or inherited according to autosomal dominant pattern of inheritance, might result in type 1B distal arthrogryposis (DA1B; MIM 614335) or in a form of congenital myopathy with tremor (congenital myopathy-16, CMYO16; MIM 618524) (Gurnett et al., 2010; Li et al., 2015; Shashi et al., 2019). To date, only 20 cases from 10 different families have been reported, all exhibiting a mild axial and mainly proximal skeletal myopathy, always associated with a distinctive postural appendicular and tongue tremor (Shashi et al., 2019; Leduc-Pessah et al., 2024; Stavusis et al., 2019; Shiraishi et al., 2022; Uneoka et al., 2023; Lanvin et al., 2024).

Here we present the clinical, molecular, and histopathological findings of a novel 38-year-old Italian CMYO16 patient.

## Methods

### Molecular studies

The study was approved by the institutional review board of the Fondazione IRCCS Ca’ Granda Ospedale Maggiore Policlinico. The patients provided written informed consent for all aspects of the study. Genomic DNA was extracted from peripheral blood samples taken from both the patient and her mother and sister. The QiaSymphony Automated Nucleic Acid Extraction Platform (QIAGEN) was utilized for this purpose.

Clinical Exome Sequencing was performed on the affected proband, starting with 100 ng of high-quality DNA. Agilent SureSelectXT Clinical Focused Exome library preparation and target enrichment kit were used. The libraries underwent paired-end sequencing on a NextSeq2000 Illumina platform. The variants included in the generated VCF files were annotated based on the genome assembly of hg19 and classified using an internal analysis pipeline and the bioinformatic tool eVai Expert Variant Interpreter v2.7.

PCR amplification followed by Sanger sequencing (using Thermo Fisher Big Dye Terminator v3.1) on an ABI Prism 3130 automated DNA analyzer was performed to validate the candidate variant in the *MYBPC1* gene in the proband and her relatives (mother and sister).

### Muscle biopsy analysis

Tissue specimens were frozen in isopentane-cooled liquid nitrogen and processed according to the standard techniques. For histological analysis, 8-µm-thick cryosections were selected and processed for routine staining with hematoxylin and eosin (H&E), modified Gomori trichrome (MGT), myosin ATPase (pH 9.4-4.6-4.3), cytochrome c oxidase (COX), succinate dehydrogenase (SDH), phosphatase acid, NADH dehydrogenase, Oil Red O, and periodic acid–schiff (PAS).

For ultrastructural examination, a small part of the muscle sample was fixed in 2.5% glutaraldehyde at pH 7.4 (Electron Microscopy Sciences EMS, Hatfield, PA, United States) for 1 h at room temperature and overnight at 4 °C; the specimens were washed in 0.1 M, pH 7.4 cacodylate buffer (EMS), and post-fixed for 1 h in 2% osmium tetroxide (EMS). Next, the specimens were dehydrated using a graded series of ethyl alcohol and embedded in Epon resin (EMS). Finally, ultrathin sections (80-nm-thick slices) were prepared using an Ultramicrotome PowerTome XL (RMC, Tucson, AZ, USA). The grids, stained with 0.5% lead citrate (EMS) and uranyl acetate replacement/methanol 1:1 (EMS), were examined using a Hitachi HT7800 Transmission Electron Microscope (Hitachi, Japan).

## Case description and results

The patient is a 38-year-old woman, nulliparous, firstborn to non-consanguineous healthy parents of Italian origin. Family history was negative for neuromuscular diseases. Main clinical events in patient’s clinical history are available in Patient’s timeline (Supplementary Table S1).

She was born at term following an uncomplicated eutocic delivery. At birth, she presented with cyanosis and neonatal hypotonia, requiring hospitalization in the Neonatal Pathology Unit. She exhibited a delay in gross motor milestone acquisition, achieving independent walking at 3 years of age. During childhood and early adolescence, she experienced fatigability, which led to the suspicion of a neuromuscular disorder. At the age of 11, a muscle biopsy was performed, and the proband recalls it revealing a partial cytochrome c oxidase deficiency; however, the results were unavailable for review. No further follow-up was conducted after, as her clinical condition remained stable. Over the years, the patient maintained a regular, non-competitive physical activity regimen, including aquatic sports and light gymnastics. However, she consistently reported pronounced fatigability even with minimal exertion, significantly greater than that of her peers, as well as a prominent postural tremor.

She came to our attention because, at the age of 37, she underwent two cycles of hormonal stimulation in the context of assisted reproductive treatment. Following the first cycle (clomiphene and follitropin beta), she developed ovarian hyperstimulation syndrome, with worsening of the fatigability, exertional dyspnea, myalgias, and a marked exacerbation of postural tremor. A second cycle (follitropin beta alone) produced similar side effects. As a result, she discontinued the physical activities she had previously maintained regularly. The exacerbation of muscle symptoms associated with hormonal stimulation gradually resolved over 6–7 months.

Current clinical findings include a high-frequency, predominantly postural tremor, most noticeable in the outstretched upper (Supplementary Video S1) and lower limbs (Supplementary Video S2). Additionally, a tongue tremor is evident following tongue protrusion (Supplementary Video S3). The tongue tremor did not impact dysarthria or dysphagia; the only speech-related “abnormality”, with uncertain relationship with tongue tremor, was isolated rhotacism. Although it is conceivable that subtle impairment of fine tongue motor control may have contributed to this articulatory feature, the clinical significance of rhotacism remains uncertain.

A mild intention tremor is also present, though less pronounced, while no rest tremor is observed. The patient also exhibits mild axial muscle weakness and a predominantly proximal weakness in the arms and legs, with a symmetric pattern. Strength examination revealed Medical Research Council (MRC) scores of 2/5 for neck flexors, 4/5 for bilateral shoulder abduction, 4+/5 for bilateral elbow flexion and extension, 4+/5 for bilateral hip flexion, 4/5 for bilateral knee flexion, 4/5 for bilateral hip adduction, and 4/5 for bilateral foot dorsiflexion, resulting in a stepping and waddling gait. On examination, a hyperlordotic posture was observed.

As regards the impact of symptoms on her life, she works as a lawyer in a predominantly sedentary setting and reported only modest impact of tremor on occupational functioning. In contrast, recreational activities were more substantially affected by early fatigability and myalgias, leading over time to spontaneous self-limitation of physical exertion.

Laboratory tests showed normal-low creatine kinase levels (29 U/L; reference range: 39–308 U/L). The electromyography (EMG) revealed diffuse mild myopathic changes in both the upper and lower limbs, characterized by a slight increase in the proportion of polyphasic motor unit potentials and a tendency for early motor unit recruitment during voluntary activation across all examined muscle groups, including the left tibialis anterior, vastus lateralis, first dorsal interosseous, and deltoid muscles. Nerve conduction studies were normal.

**FIGURE 2:**
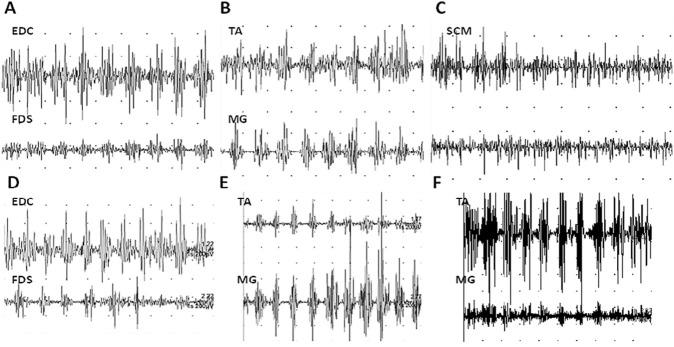
EMG recordings of tremor. In **(A,B)** two-channel polygraphic recordings from antagonist muscles, showing the coactivation pattern of antagonist muscles. In A, signals were recorded from the left extensor digitorum communis (EDC) and flexor digitorum superficialis (FDS) while the patient maintained the arm extended forward with the palm facing downward in a neutral position between flexion and extension. In B, recordings were obtained from the right tibialis anterior (TA) and medial gastrocnemius (MG) while the patient was standing. In **(C)** EMG recording of tremor activity in the right sternocleidomastoid (SCM) muscle while the patient maintained the head rotated to the left. In **(D)** EMG recording from the left EDC and FDS during loading test (0.5 kg). In **(E,F)** recordings obtained from the right TA and MG during tiptoe standing **(E)** and heel standing **(F)**. Across all recordings, tremor frequency remains constant at approximately 10–11 Hz. The horizontal axis represents time, with each division corresponding to 1 s. The vertical axis represents signal amplitude, with a scale of 200 µV/div in **(A,B)**, and 1 mV/div in **(C)**.

A muscle MRI of the lower limbs was performed, revealing mild global muscle hypotrophy, without fatty replacement or muscle edema. Pulmonary evaluation revealed a moderate restrictive ventilatory defect with a reduction in expiratory volumes. Forced vital capacity (FVC) in the seated position was 2.25 L (58% of predicted), and forced expiratory volume in 1 s (FEV1) was 2.15 L (68% of predicted). Maximal Inspiratory Pressure was 36 cmH_2_O (42% predicted) and maximal expiratory pressure was 33 cmH_2_O (34% predicted). Polisonnography was normal, as was blood gas analysis. Polygraphic tremor analysis (Figure 2) confirmed the absence of rest tremor and the presence of a postural tremor with antagonist muscle coactivation at a frequency of 10–11 Hz, affecting both the upper limbs and lower limbs. In the upper limbs, the loading test resulted in an amplitude increase without frequency modification. Similarly, in the lower limbs, muscle activation (tiptoe or heel standing) led to an increased EMG burst amplitude at the activated muscle, without changes in frequency. EMG of the right masseter and sternocleidomastoid (SCM) muscles showed electrical silence at rest, with a tendency toward tremor activation at ∼10 Hz during muscle contraction.

Cardiac evaluation, including echocardiography and 24-h Holter ECG monitoring, was unremarkable, with a preserved left ventricular ejection fraction of 76% and no relevant abnormalities detected.

**FIGURE 3:**
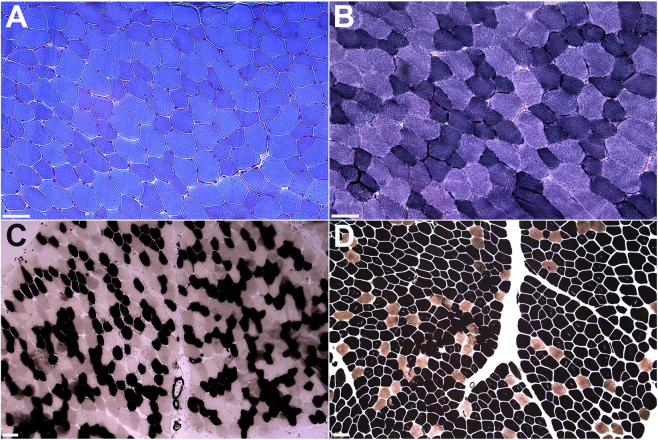
Muscle histology and histochemistry. Modified Gomori trichrome **(A)**, NADH-TR **(B)**, pH 4.3 **(C)** and 4.6 **(D)** myosin ATPase stains showing type I fiber predominance and hypotrophy. Scale bar = 50 μm.

**FIGURE 4:**
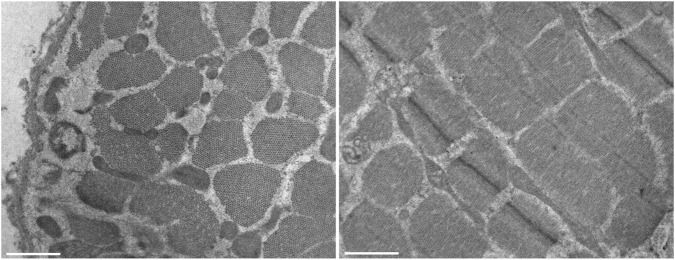
Muscle ultrastructural analysis. Electron microscopy images showing normal myofibrillar architecture, rare mitochondria of elongated shape. Scale bar = 1 μm.

Muscle biopsy of the biceps brachii muscle demonstrated a predominance of type I fibers, which appeared smaller than type II fibers (Figure 3). Ultrastructural examination did not reveal any alterations characteristic of congenital myopathies, but showed normal myofibrillar architecture, rare mitochondria of elongated shape, and mild subsarcolemmal increase in glycogen content (Figure 4).

Clinical exome sequencing revealed the presence of the heterozygous c.788T>G p.(Leu263Arg) variant in exon 11 of the *MYBPC1* gene. The variant was confirmed by Sanger sequencing in the proband, whereas it was absent in the patient’s healthy mother and sister. Proband’s father was not available for segregation testing, precluding the definitive confirmation of a *de novo* origin for the variant. The p.Leu263Arg variant was previously described in the literature (Shashi et al., 2019) and classified as likely pathogenic (class 4) according to the ACMG (American College of Medical Genetics and Genomics) criteria.

Receiving a molecular diagnosis had a somewhat positive impact, as it provided a unifying explanation for her longstanding symptoms. In the background of expression variability, *MYBPC1* molecular defects leading to CMYO16 are inherited *de novo* or with autosomal dominant inheritance and display an almost complete penetrance. Therefore, the risk for a patient harbouring the molecular defect of transmitting the disease to offspring is 50%. Most of the molecular defects so far identified are single nucleotide variants that can be easily identified by prenatal testing or Preimplantation Genetic Testing for monogenic disorders (PGT-M) in medically assisted procreation approaches.

Despite counselling regarding the possibility of prenatal diagnosis, she opted against further assisted reproductive treatment, in order to avoid renewed hormonal stimulation and possible clinical worsening, and she is currently pursuing adoption. In addition to avoiding further hormonal stimulation, at this time she was also reluctant to pursue a spontaneous pregnancy, had it ever occurred, because of concern that gestation could further impair respiratory function in the setting of restrictive ventilatory defect.

## Discussion and conclusion

We report the case of a young woman presenting with mild axial and proximal weakness, associated with a distinctive high-frequency postural tremor affecting the limbs and tongue. Laboratory investigations showed normal-low CK levels. Muscle MRI was substantially normal. EMG revealed diffuse mild myopathic changes. Muscle biopsy findings, documenting a selective type 1 fiber hypotrophy, was consistent with a congenital myopathy. The detection of the p.Leu263Arg pathogenetic variant in the *MYBPC1* gene allowed the achievement of a definitive diagnosis of CMYO16. Although family history for neuromuscular disorders is negative, incomplete parental segregation analysis limits the definition of the variant as *de novo* or presumed *de novo*.

Heterozygous damaging *MYBPC1* variants have been associated with distal arthrogryposis (MIM 614335) and an early‐onset congenital myopathy with tremor (MIM 618524) (Shashi et al., 2019; Leduc-Pessah et al., 2024; Stavusis et al., 2019; Shiraishi et al., 2022; Uneoka et al., 2023; Lanvin et al., 2024). To date, only a limited number of families with heterozygous *MYBPC1*-related myopathy and tremor have been reported (Supplementary Table S2). Pediatric and adult-onset patients have been reported, indicating that the disease can manifest across a broad age spectrum (Uneoka et al., 2023). The clinical course is generally mild and slowly progressive, with tremor often representing the most disabling symptom. The associated tremor is present from early childhood and remains relatively stable over time.

Affected individuals typically present with early-onset hypotonia, delayed motor development, generalized muscle weakness, and a high-frequency postural tremor thought to be of myogenic origin. Tongue tremor with fasciculations is a frequent hallmark, characterized by irregular, undulating waves reflecting localized tongue twitching. In our patient, the tremor was irregular and wavelike, consistent with previous reports (Shashi et al., 2019; Stavusis et al., 2019; Geist Hauserman et al., 2021). In contrast to our patient, some individuals may also present with skeletal deformities such as scoliosis, thoracic asymmetry, and mild facial dysmorphia (Stavusis et al., 2019; Uneoka et al., 2023). Overall, the phenotype observed in our patient aligns with these previously described features, highlighting mild myopathy with tremor as the predominant functional limitation. In our patient, respiratory involvement deserves further consideration. Our patient had presented neonatal cyanosis and hypotonia. Neonatal cyanosis, although not initially interpreted as neuromuscular in origin, may retrospectively suggest early respiratory dysfunction. Recent reports (Lanvin et al., 2024) have emphasized neonatal respiratory distress and stridor as part of the *MYBPC1* phenotypic spectrum. In our patient, a moderate restrictive ventilatory defect identified in adulthood supports the presence of persistent but clinically subtle respiratory involvement. The apparent clinical stability after childhood, with no medical attention sought until adulthood, may in part reflect under recognition of symptoms, possibly related to her sedentary lifestyle. These findings suggest that respiratory dysfunction in *MYBPC1*-related myopathy may be present early but remain clinically silent, highlighting the need for systematic respiratory evaluation.

The presence of mild myopathy suggests a dosage-dependent role of *MYBPC1* in muscle pathology. Knock-in mouse models carrying the E248K variant (E248K-KI mouse model) demonstrate that partial loss of sMyBP-C function leads to sarcomeric disorganization, reduced force generation, and features of mild myopathy and tremor (Geist Hauserman et al., 2021). In contrast, homozygous loss-of-function variants result in absent protein expression and severe or lethal phenotypes, likely due to fetal akinesia *in utero* (Markus et al., 2012; Ekhilevitch et al., 2016; Gurnett et al., 2010; Li et al., 2015).

Heterozygous *MYBPC1* variants are consistently associated with tremor. Stavusis et al. were the first to propose a myogenic origin for this kind of tremor (Stavusis et al., 2019). They described a high-frequency postural tremor in all affected individuals, involving the arms and legs (p.E248K; p.Y247H), and, in some cases, the tongue (p.Y247H). Similarly to our case, Fourier analysis revealed a 10–11 Hz postural tremor with co-contraction of agonist and antagonists, that remained unchanged with weight loading. The absence of tremor frequency change after weight loading of the limbs might indicate a dysfunction in the oscillating neural networks within the central nervous system, suggesting the existence of a pacemaker at the origin of the tremor, although it does not define the location (central versus peripheral) of the pacemaker.

Asynchronous tremor activity between different body segments, as well as between different sides of the same body segment (Video) suggested the involvement of multiple tremor generators. These findings closely align with the results of our patient’s polygraphic tremor analysis and are consistent with other clinical descriptions of this tremor (Shashi et al., 2019; Shiraishi et al., 2022). Although the tremor frequency remains stable under weight loading, *MYBPC1* is exclusively expressed in skeletal muscle and is absent from the central nervous system and peripheral nerves, making a primary central origin unlikely (Stavusis et al., 2019). Instead, the authors proposed a sarcomeric origin, where the mutant sMyBP-C leads to dysregulated cross-bridge cycling, causing not only a mild force deficit but also an oscillatory contraction pattern upon muscle activation. This oscillation is then picked up by stretch receptors and propagated through a sensorimotor feedback loop, leading to a centrally modulated, but sarcomeric-initiated, tremor (Stavusis et al., 2019). This mechanism has been recently investigated in the *Mybpc1* E248K-KI model. Compared to control littermates, soleus myofibres of E248K-KI animals showed delayed increase in force after a rapid stretch. This myofilament stretch activation was induced in mutant animals as a likely consequence of spontaneous pulsatile sarcomere oscillations in a subset of myofibres, after submaximal calcium activation. The resulting sinusoidal waveform pattern in sarcomere length often persisted for minutes (Mariano et al., 2025a). These findings suggest that heterozygous pathogenetic molecular defects in *MYBPC1* might compromise the role of sMyBP-C in the regulation of synchronous activation of myofilaments by exacerbating both spontaneous oscillatory activity and stretch-dependent activation.

Myogenic tremor, a clinical entity named “myotremor” (Schaefer et al., 2021), has been described in other congenital myopathies, all linked to mutations in genes encoding sarcomere-associated proteins, including both thick filament proteins (*MYH7*, *MYH2*, *MYL2*) and thin filament proteins (*TNNT1*, *NEB*, *TPM3*, *TTN*) (Stavusis et al., 2020). In this context it should be noted that the almost exclusive expression of these genes in skeletal muscle is the main finding supporting the localization of the tremor peacemaker to the sarcomere.

Two *MYBPC1* missense mutations, the p.E248K and p.Y247H, have been associated with a dominant, mild skeletal myopathy invariably accompanied by myotremor and resulting from altered sarcomeric function due to the *MYBPC1* mutations. These two mutations are in the N-terminal M-motif of sMyBP-C and result in markedly increased binding of the N-terminus to myosin, possibly interfering with normal cross-bridge cycling as the first muscle-based step in tremor genesis (Stavusis et al., 2019).

Our patient carries the previously described likely pathogenic heterozygous c.788T>G p.(Leu263Arg) (L263R) variant in exon 11 of the *MYBPC1* gene (Shashi et al., 2019). Functional expression studies by Shashi et al. demonstrated that the L263R mutation results in a significantly reduced binding of the M-motif to myosin compared with the wild type, likely impairing the formation of actomyosin cross-bridges during muscle contraction. Binding to actin was comparable to that of the wild type (Shashi et al., 2019). All pathogenic variants described to date, including the one shared with our patient, appear to be located within the M-motif in the N-terminus of the sMyBP-C protein.

The reproducible clinical worsening observed after ovarian stimulation in our patient is of particular interest. Sex hormones are known to influence clinical presentations across several neuromuscular disorders (Vinciguerra et al., 2023). Although a direct link between *MYBPC1* variants and hormonal responsiveness is not established, and sex differences in the phenotype is not reported in humans, this observation raises the possibility that endocrine stress may modulate symptom expression in *MYBPC1*-related myopathy. Indeed, interestingly, recent studies in animal models suggest that *MYBPC1*-related phenotypes may be modulated by sex (Mariano et al., 2025b). Such findings cannot be directly extrapolated to humans, but our observation may suggest that symptom expression in *MYBPC1*-related myopathy may be sensitive to endocrine perturbations rather than to sex alone.

In line with previously reported literature, the muscle biopsy of our patient also revealed mild myopathic changes, including fiber size variability and type I fiber predominance and hypotrophy (Stavusis et al., 2019). These histological features are relatively nonspecific and can be observed across various congenital myopathies, including those caused by mutations in sarcomeric proteins. The predominance expression of *MYBPC1* in type 1 fibers likely explains the selective involvement of slow-twitch fibers. Muscle biopsies from DA1 due to *MYBPC1* defects also demonstrated type I fiber atrophy (Gurnett et al., 2010).

As of now, there is no specific cure for *MYBPC1*-associated myopathy. Management is primarily supportive, focusing on physical therapy to maintain muscle strength and function, and symptomatic treatment of the tremor if it significantly impacts daily activities. In our patient, no symptomatic treatment for tremor was attempted, considering the available literature suggesting limited or no benefit of primidone and beta-blockers in this form of tremor, as well as concern regarding their potential respiratory side effects. Further research is needed to explore targeted therapies that address the underlying molecular mechanisms of the disease.

As regards myalgias, for 3 months she was treated with duloxetine 30 mg/day, which she reported to be beneficial, but this was subsequently discontinued because of gastrointestinal hyperactivity. The subjective improvement in myalgias observed with duloxetine may be interpreted in the context of its established analgesic effects on central pain modulation, rather than as evidence of a specific therapeutic effect on the underlying sarcomeric defect. By contrast, the bowel symptoms leading to discontinuation are plausibly attributable to the known gastrointestinal adverse effects of SNRIs, for which no *MYBPC1*-specific susceptibility has been described to date.

Our patient is currently undergoing a respiratory physiotherapy program and is followed with 6-monthly neurological and respiratory assessments.

At 1-year follow-up after diagnosis, her condition remains clinically stable from both the motor and respiratory perspectives. Indeed, after adolescence (around the age of 11), the patient had not sought further medical attention until adulthood, following ovarian stimulation, giving the impression of a relatively stable disease course. This observation supports the hypothesis that *MYBPC1*-related myopathy is present from early life but may remain clinically silent or underestimated for long periods, only becoming evident under conditions of increased physiological stress.

Finally, it is crucial to recognize that tongue fasciculations, which can be difficult to distinguish from tremor without electrophysiological data, delayed motor development, and early-onset hypotonia are common features observed in central or peripheral nervous system disorders that must be considered for differential diagnoses (Uneoka et al., 2023). For example, sarcomeric congenital myopathies often display infantile onset of muscle weakness, feeding difficulties, and respiratory issues (i.e., nemaline myopathies). Similarly, congenital muscular dystrophies commonly show early onset muscle weakness and delayed motor milestones (Stavusis et al., 2020).

Most importantly, tongue fasciculation and hypotonia are highly suggestive of spinal muscular atrophy (SMA), a condition for which multiple effective therapies, including gene therapy, are currently available (Klotz et al., 2021). Therefore, the discrimination of SMA-related tongue fasciculations from *MYBPC1*-associated abnormal (undulating) tongue movements is important for a rapid identification of SMA subjects. Once SMA has been excluded by diagnostic genetic testing or negative results of newborn screening (wherever available), alternative phenotypes, including *MYBPC1*-related myopathy should be considered and addressed by using extended (NGS-based) molecular screening in presence of early onset muscle weakness.

## Data Availability

The original contributions presented in the study are included in the article/Supplementary Material, further inquiries can be directed to the corresponding authors.
