# Supplementary material for: MYBPC1-associated congenital myopathy with tremor: further delineation of the clinical and pathological phenotype in the first Italian case
Source: Front Genet. 2026 May 14;17:1809063. doi: 10.3389/fgene.2026.1809063 (PMC13215647; doi:10.3389/fgene.2026.1809063)
Supplement: Supplementary file 1 [file DataSheet2.pdf]

Table 2. Clinical and Genetic Features Associated With *MYBPC1* Variants

Abbreviations: F, female; M, male; N, normal; y, years; m, months; Fatig, fatigability ; SW, scapular winging; HAP, high arched palat; micro, microretrognathia; IQ, intelligence quotient; n.a , data not available

Reference	Our case	Lanvin et al., 2024			Uneoka et al., 2023				Shirashi et al., 2022	Stavusis et al., 2019								Shashi et al., 2019			
**MYBPC1 variant**	c.788T>G, p.(L263R)	c.742G>A; p.(E248K)	c.788T>G, p.(L263R)	c.788T>G, p.(L263R)	c.788T>G, p.(L263R)	c.742G>A, p.(E248K)	c.742G>A, p.(E248K)	c.742G>A, p.(E248K)	c.788T>G, p.(L263R)	c.742G>A, (E248K)	c.742G>A, p.(E248K)	c.742G>A, p.(E248K)	c.742G>A, p.(E248K)	c.739T>C, p.(Y247H)	c.739T>C, p.(Y247H)	c.739T>C, p.(Y247H)	c.739T>C, p.(Y247H)	c.788T>G, p.(L263R)	c.788T>G, p.(L263R)	c.788T>G, p.(L263R)	c.776T>C, p.(L259P)
**Familial/Sporadic**	S1	S2	F1	F2	S3	F3	F4	F5	S4									S5	F6	F7	S6
**Inheritance**	***De novo***	***De novo***	*Paternally inherited*	*Father of F1*	***De novo***	*Maternally inherited*	*Maternally inherited (sister of F3)*	*Maternally inherited (mother of F3)*	*De novo*									*De novo*	*Paternally inherited*	*Father of F6*	*De novo*
**Protein domain**	M motif	M motif	M motif	M motif	M motif	M motif	M motif	M motif	M motif	M motif	M motif	M motif	M motif	M motif	M motif	M motif	M motif	M motif	M motif	M motif	M motif
**Gender/age**	**F / 38 y**	**F / 32 mo**	**M / 9 y**	**M / n.a**	**F / 5 y**	**F / 10 y**	**F / 5 y**	**F / 36 y**	**F/ 27y**	**F / 57 y**	**M / 28 y**	**M / 30 y**	**F / 5 y**	**F / 50 y**	**M / 32 y**	**F /3 y**	**M / 6 m**	**F / 9 y**	**F / 9 y**	**M / 40 y**	**M / 23 m**
**Age at onset**	**At birth**	**At birth**	**At birth**	**At birth**	**5 m**	**7 m**	**6 m**	**5 m**	**At birth**			**Since infancy**		**Since childhood**				**At birth**	**At birth**	**At birth**	**At birth**
**Delay of motor development**	**+**	**+**	**+**	**+**	**+**	**+**	**+**	**+**	**+**			**+**					**+**	**+**	**+**	**+**	**+**
**Mental development**	**N**				**N**	**IQ 65**	**IQ 88**	**N**	**N**	**N**	**N**	**N**	**N**	**N**	**N**	**N**	**N**	**N**	**N**	**N**	**N**
**Postural tremor**	**+**	**+**	**+**	**+**	**+**	**+**	**+**	**+**	**+**	**+**	**+**	**+**	**+**	**+**	**+**	**-**	**+**	**+**	**+**	**+**	**+**
**Tongue tremor**	**+**	**Jaw tremor**			**+**	**+**	**+**	**+**	**+**					**+**	**+**	**+**		**+**	**+**	**-**	**+**
**Contractures**	**-**		**+**						**+**	**+**	**+**	**+**	**+**	**+**	**+**	**-**	**+**	**-**	**-**	**-**	**-**
**Muscle biopsy/EMG**	**+/+**	**n.a/-**							**+/+**	**-/+**	**-/-**	**-/-**	**-/-**	**+/+**	**-/-**	**-/-**	**-/-**				
**Hypotonic face**	**-**				**+**	**-**	**-**	**-**	**+**	**-**	**-**	**-**	**-**	**-**	**-**	**-**	**-**	**-**	**+**	**+**	**-**
**Scoliosis**	**-**				**-**	**Very mild**	**-**	**-**	**+**	**+**	**+**	**+**	**-**	**Mild**	**Mild**	**-**	**-**	**-**	**-**	**-**	**-**
**Others**	**Fatig**		**Mild micro, Darwininan nodule right ear**	**Fatig**					**Fatig, SW, HAP**					**SW, HAP, cramp**	**SW, HAP**	**SW, HAP**			**SW, epilepsy**	**Fatig**	
