# Supplementary material for: MYBPC1-associated congenital myopathy with tremor: further delineation of the clinical and pathological phenotype in the first Italian case
Source: Front Genet. 2026 May 14;17:1809063. doi: 10.3389/fgene.2026.1809063 (PMC13215647; doi:10.3389/fgene.2026.1809063)
Supplement: Supplementary file 2 [file DataSheet1.pdf]

Table 1

Timeline of the patient’s clinical history, diagnostic work-up. ART: Assisted Reproductive Technology; CES: clinical exome sequencing analysis

**Onset of symptoms**	**Motor development**	**First symptoms**			
**At birth**	**3 years**	**Childhood and early adolescence**	**11 years**	**11-37 years**	**37 years**
					2 Hormonal stimulations (ART)
Cyanosis and neonatal hypotonia (hospitalization in the Neonatal Pathology Unit)	Walked independently	Fatigability and postural tremor	1st muscle biopsy: partial cytochrome c oxidase deficiency – report unavailable	No further follow-up; clinical condition stable: persistent fatigue and postural tremor	**Fatigue, dyspnea, myalgia, postural tremor ↑**<br>- Neurological examination: postural tremor upper and lower limbs, tongue tremor, mild intention tremor, mild proximal upper and lower limbs and axial weakness, hyperlordotic posture<br>- Normal CPK levels; EMG: mild myopathic pattern; muscle MRI: mild global hypotrophy, restrictive ventilatory defect at spirometry; 2nd muscle biopsy: type I fiber hypotrophy
					**CES: c.788T>G, p.(L263R) in MYBPC1**
